# Bacteria spring a surprise

**DOI:** 10.7554/eLife.03435

**Published:** 2014-06-24

**Authors:** Ramanujam Srinivasan, Mohan K Balasubramanian

**Affiliations:** 1 **Ramanujam Srinivasan** is in the Centre for Biosciences and Biomedical Engineering, Indian Institute of Technology, Indore, Indiakuruhoornambi@gmail.com; 2 **Mohan K Balasubramanian** is at the Warwick Medical School, University of Warwick, Coventry, United Kingdom, and the Temasek Life Sciences Laboratory, the Department of Biological Sciences and the Mechanobiology Institute, National University of Singapore, Singapore, Singaporem.k.balasubramanian@warwick.ac.uk

**Keywords:** Caulobacter crescentus, Par system, DNA partitioning, chromosome dynamics, intracellular transport, other

## Abstract

Elastic forces within DNA drive the segregation of chromosomes in bacteria.

**Related research article** Lim HC, Surovtsev IV, Beltran BG, Huang F, Bewersdorf J, Jacobs-Wagner C. 2014. Evidence for a DNA-relay mechanism in ParABS-mediated chromosome segregation. *eLife* **3**:e02758. doi: 10.7554/eLife.02758

**Image** Partition complexes (red arrows) change shape as chromosomes segregate in bacteria

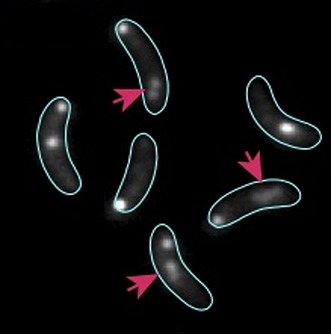

Cell division involves the precise duplication of all the genetic material in the cell—both the chromosomes and the plasmid DNA—and the equal division of this material between the two daughter cells. Eukaryotes employ a structure called the mitotic spindle, which is made of microtubules, and various motor proteins to ensure proper chromosome segregation (Walczak and Heald, 2008). Bacteria, meanwhile, rely on actin-like or tubulin-like proteins to segregate plasmid DNAs (Salje et al., 2010; Oliva et al., 2012), but little is known about the segregation of chromosomes in bacteria.

In 1963, François Jacob and colleagues suggested a passive mechanism in which DNA segregation was coupled to cell elongation (Figure 1A). Although several other mechanisms have been proposed since then, the molecules that orchestrate chromosome segregation in bacteria and the details of the segregation process are only beginning to emerge.

**Figure 1.**
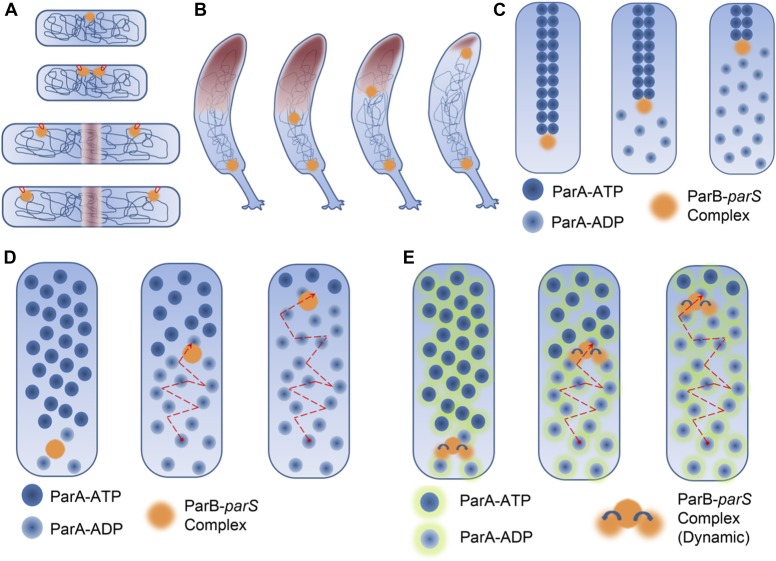
DNA segregation in bacteria. (**A**) In the membrane tether model (Jacob et al., 1963), DNA replication begins at a DNA sequence called the origin of replication (orange circle; top). After replication, there are two origins of replication, both of which are tethered to specific sites on the membrane (tethers shown in red). Cell elongation pulls the two origins of replication away from the centre of the cell, and this passively divides the DNA between the daughter cells. (**B**) During chromosome segregation in *C. crescentus*, one of the ParB-*parS* partition complexes (orange circles) remains at the original pole, while the other partition complex appears to be pulled towards the new pole at the opposite end of the cell by a cloud of the ParA enyzme (brown shading). (**C**) In the pulling model for DNA segregation, ParA bound to ATP can form a cytoskeletal filament (dark blue spheres), and the ParB in the partition complex (orange circle) can stimulate hydrolysis of the ParA-ATP to form ParA-ADP (light blue spheres): this causes one end of the filament to depolymerise. The partition complex remains attached to the filament, so it is pulled to one end of the cell as the filament undergoes depolymerisation, as are half of the chromosomes (not shown) in the cell. (**D**) In the diffusion-binding model the partition complex (and anything attached to it) can also end up at one pole of the cell as a result of diffusion. As in the pulling model shown in (**C**), this model relies on ParB stimulating the hydrolysis of ParA-ATP; overall the partition complex diffuses towards higher concentrations of ParA (Vecchiarelli et al., 2010). (**E**) In the DNA relay mechanism proposed by Lim et al., the ParB-*parS* complex (orange) attaches to ParA-ATP (dark blue spheres with green halos) bound to DNA and moves as a result of being stretched by the elastic forces of the chromosome (not shown), before the ParB stimulates the hydrolysis of ParA-ATP to form ParA-ADP (light blue spheres with green halos).

Now, in *eLife*, Christine Jacobs-Wagner and co-workers at Yale—including Hoong Chuin Lim as first author—describe a novel mechanism by which the bacterium *Caulobacter crescentus* segregates its chromosomes through the use of a DNA-protein complex called the ParABS complex (Lim et al., 2014). In an important deviation from other models, the mechanism proposed by the Yale team relies on the elastic nature of the chromosome and a protein gradient formed by an enzyme in the ParABS complex.

This complex is made by an enzyme called ParA and a 'partition complex' that is formed by a protein (ParB) binding to a length of DNA called *parS* (Gerdes et al., 2010; Mierzejewska and Jagura-Burdzy, 2012). Although the ParABS complex was first studied because of its role in plasmid maintenance, the *parS* sequence has been found in the vicinity of a DNA sequence called the origin of replication in many different bacteria (Livny et al., 2007). In *C. crescentus*, prior to DNA replication, the ParA enzyme and the ParB-*parS* partition complex are spatially segregated: the partition complex resides at one end (or pole) of the cell, while the enzyme spreads out in a 'cloud' from the other end of the cell (Schofield et al., 2010). Following DNA replication, there are two partition complexes: one remains where it was, while the other appears to be pulled towards the opposite pole by the cloud of ParA (Figure 1B).

Several in vitro and in vivo studies have presented evidence that ParA, when bound to a molecule of ATP, can assemble into filaments: moreover, ParB can cause these filaments to break down by stimulating the hydrolysis of ATP (Dye and Shapiro, 2007; Ptacin et al., 2010; Szardenings et al., 2011). These observations have led to a pulling model of DNA segregation in bacteria (Figure 1C). This model is similar in some ways to the spindle mechanism in eukaryotes in that it involves the assembly and disassembly of filamentous structures.

A twist in this tale was the proposal of a diffusion-binding model for plasmid DNA segregation (Figure 1D). In this model, the partition complex diffuses through the cell and binds to ParA-ATP that is already bound to DNA. The ParB in the complex then stimulates the hydrolysis of the ATP, which results in the release of the complex. The complex then moves to a region where there is a higher concentration of ParA bound to DNA, and the whole process is repeated. The end result is that the partition complex (and any plasmid DNA attached to it) keeps moving in one direction.

The results of the Yale team, however, are not consistent with these models. Careful biochemical experiments revealed that although ParA formed dimers, it did not form multimers, and super resolution microscopy showed that it is spread out in the cell in a way that is inconsistent with the presence of ParA filaments. Time-resolved microscopy also revealed that the partition complex moved in a way that is inconsistent with the existence of ParA filaments. The results of computational simulations, meanwhile, were not consistent with the diffusion-binding model.

How, then, are the chromosomes in *C. crescentus* segregated? Lim et al. observed that three different positions on the chromosomes exhibited dramatic fluctuations in their position, both in the long and short axes of the cell. In light of this observation, they considered the possibility that elastic forces within the DNA molecules themselves (modelled as springs) might power the segregation of chromosomes. Through a series of computations, using a number of experimentally derived parameters, they estimated the spring constant for chromosomes, as well as the magnitude of the forces acting on the partition complex.

These data and additional simulations led Lim et al. to propose the DNA relay mechanism (Figure 1E). In short, the partition complex is captured by ParA-ATP dimers that are bound to DNA. Forces exerted by the DNA stretch the complex, and by the time that hydrolysis has taken place, the complex has been pulled into a new position. Hydrolysis of ATP subsequently releases the complex. This hydrolysis also causes a lower concentration of ParA-ATP dimers in the vicinity of the partition complex and this results in the formation of the ParA protein gradient, with the highest concentrations of ParA-ATP being found at the new cell pole away from the partition complex. The partition complex is again captured by ParA-dimers bound to DNA. This results in the complex moving away from the old cell pole and towards the new cell pole. A repetition of these steps eventually leads to chromosome segregation.

Lim et al. carefully measured the aspect ratio of the partition complex and found that the value of this ratio measured during the process of segregation was different to that measured afterwards. Importantly, while undergoing segregation, the partition complex exhibited a stretched conformation that was consistent with the force being exerted by the DNA.

In summary, the Yale team has proposed an exciting new mechanism for DNA segregation in which the forces that drive segregation are generated by the elastic properties of the DNA itself, rather than being generated by the cytoskeleton or molecular motors. The model also generated important predictions that were confirmed by experiments. It will be interesting to see how widespread such a mechanism may be, both within bacteria and beyond.
